# AKT1 restricts the invasive capacity of head and neck carcinoma cells harboring a constitutively active PI3 kinase activity

**DOI:** 10.1186/s12885-018-4169-0

**Published:** 2018-03-05

**Authors:** Sanja Brolih, Scott K. Parks, Valérie Vial, Jérôme Durivault, Livio Mostosi, Jacques Pouysségur, Gilles Pagès, Vincent Picco

**Affiliations:** 1 Centre Scientifique de Monaco, Department of Medical Biology, 8 Quai Antoine Ier, Monaco, Principality of Monaco; 2 UCA, Université Côte d’Azur, Nice-Sophia-Antipolis, Institute for Research on Cancer and Aging of Nice, CNRS-UMR 7284-Inserm U1081, Nice, France

**Keywords:** Cancer, Metastasis, AKT/PKB, Head and neck squamous cell carcinoma, Phenotypic screening, Epithelial-mesenchymal transition, Adhesion, Migration, Invasion

## Abstract

**Background:**

In mammals, the AKT/PKB protein kinase family comprises three members (AKT1–3). PI3-Kinase (PI3K), a key oncogene involved in a wide variety of cancers, drives AKT activity. Constitutive activation of the PI3K/AKT pathway has been associated with tumorigenic properties including uncontrolled cell proliferation and survival, angiogenesis, promotion of cellular motility, invasiveness and metastasis. However, AKT1 activity has also been recently shown to repress the invasive properties of breast cancer cells in specific contexts.

**Methods:**

This study used both pharmacological and shRNA approaches to inhibit AKT function, microscopy to characterize the cellular morphology, 3D spheroid models to assess migratory and invasive cellular capacities and a phenotypic screening approach based on electrical properties of the cells.

**Results:**

Here we demonstrate that the alternative action of AKT1 on invasive properties of breast cancers can be extended to head and neck carcinomas, which exhibit constitutive activation of the PI3K/AKT pathway. Indeed, inhibition of AKT1 function by shRNA or a specific pharmacological inhibitor resulted in cellular spreading and an invasive phenotype. A phenotypic screening approach based on cellular electrical properties corroborated microscopic observations and provides a foundation for future high-throughput screening studies. This technique further showed that the inhibition of AKT1 signaling is phenocopied by blocking the mTORC1 pathway with rapamycin.

**Conclusion:**

Our study suggests that the repressive action of PI3K/AKT1 on cellular invasive properties may be a mechanism common to several cancers. Current and future studies involving AKT inhibitors must therefore consider this property to prevent metastases and consequently to improve survival.

**Electronic supplementary material:**

The online version of this article (10.1186/s12885-018-4169-0) contains supplementary material, which is available to authorized users.

## Background

The serine/threonine kinase AKT promotes the epithelial-mesenchymal transition (EMT), cellular motility and invasion in a wide variety of physiological and pathological conditions. In developing vertebrate embryos, the EMT events leading to the formation of mesoderm during gastrulation rely on AKT activity and its upstream activator phosphatidylinositol 3-kinase (PI3K) [1–3]. AKT activity is also associated with EMT, migration and invasion in a diverse range of tumor cell models [4–6]. Furthermore, AKT is a major mediator of cell survival through direct inhibition of pro-apoptotic proteins. Based on these observations, AKT was proposed to be a suitable target for anticancer therapies and clinical trials using inhibitors of PI3K or AKT have been performed previously and are on-going [7].

Among the most recent clinically suitable AKT inhibitors, MK-2206, an allosteric inhibitor targeting AKT isoforms 1–3 with an affinity in the nanomolar range, has undergone phase II clinical trials in several cancer types [8–11]. Despite acceptable tolerance and promising preclinical results, none of the clinical trials have shown favorable effects for MK-2206 treatment [8–24]. Interestingly, the different AKT isoforms can display drastically different functions, especially with regards to cell migration. For example, preclinical data suggested that AKT2 promotes metastasis in ovarian and breast cancer cell models [25, 26] while AKT1 was proposed to inhibit the migration and metastasis of breast cancers [27–30]. Collectively, these results raise concerns over the clinical outcomes of pan-AKT inhibitors and indicate the potential benefits of pursuing isoform-specific inhibitors.

In the present study, we extend recent results showing that AKT1 restricts the invasive potential of breast tumor cells [27–30] to head and neck squamous cell carcinoma (HNSCC) cells. In these HNSCC model cell lines, AKT1 inhibition induced a drastic change in the cellular morphology of the CAL33 oral cancer cell line that is associated with increased migratory and invasive capacities. By means of phenotypic screening based on electrical cellular properties, we identified other HNSCC cell lines that either demonstrated the same phenotype (Detroit562) or an absence of drug sensitivity (CAL27). We next screened for other anti-cancer compounds and showed that the mTORC1 inhibitor rapamycin induces a comparable modification of the cellular electrical properties and modification of CAL33 and Detroit562 cell morphology. Our results therefore extend the concept that AKT1 exerts an anti-metastatic effect. Finally, our results suggest that some anti-cancer drugs may induce a pro-metastatic effect and proposes an efficient in vitro screen to evaluate this effect. This unexpected detrimental effect of anti-cancer drugs should be considered with caution and emphasizes the need for personalized therapies.

## Methods

### Cell lines, shRNAs and pharmacological inhibitors

All cell lines used in this study were cultured in Dulbecco’s Modified Eagle’s Medium (DMEM, Invitrogen) supplemented with 7.5% heat-inactivated fetal calf serum (FCS) at 37 °C in an atmosphere of 5% CO_2_.The three human head and neck cancer cell lines used in this study, CAL33, CAL27 and Detroit562 were provided through a Material Transfer Agreement with the Oncopharmacology Laboratory, Centre Antoine Lacassagne (Nice, France) where they had initially been isolated [31]. For knock-down experiments, the cells were infected using a lentiviral vector containing non-target control or two independent sequences of shRNA targeting the product of the AKT1 gene (shAKT1.1 5′- GGACAAGGACGGGCACATTAA -3′ and shAKT1.2 5′- CTATGGCGCTGAGATTGTGTC -3′) cloned in pLKO.1 puro, (gift from Bob Weinberg, Addgene plasmid #8453) according to the protocol available at www.addgene.com. A total population of cells was generated by selection with 10 μg/mL puromycin for 10–15 days. MK-2206 (S1078, Selleck Chemicals) AKT inhibitor was used at a final concentration of 5 μM. Rapamycin (S1039, Sellek Chemicals) mTORC1 inhibitor was used at a final concentration of 5 μM. Erlotinib (S7786, Selleck Chemicals) EGFR inhibitor was used at a final concentration of 5 μM.

### Phalloidin staining and e-cadherin immunostaining

Cells were grown at low density on glass coverslips or 24-well plates and fixed with PBS-4% paraformaldehyde (PFA) for 10 min followed by saturation for 30 min in PBS-3% skim milk-0.1% Triton X-100. Cells were then stained with phalloidin actin-stain 555 (Euromedex) according to the manufacturer’s protocols. In certain preparations, immunostaining was then performed afterwards by incubating the cells overnight at 4 °C in the dark in a 1/100 dilution of an e-cadherin antibody (14,472, Cell Signaling Technology). Cells were then washed 3 times in PBS, incubated for 2 h at room temperature in the dark in a 1/500 dilution of an anti-mouse Alexa Fluor 488 conjugated antibody (4408, Cell Signaling Technology) and washed 3 times in PBS. The nuclear DNA was counterstained with Hoechst 33,342 (4082, Cell Signaling Technology) according to the manufacturer’s protocols. Cells grown on coverslips were mounted onto slides with Prolong Gold Antifade moutant (Thermo Fischer Scientitifc) and images were captured using a Leica DMI-4000 microscope (Leica Microsystems) equipped with a Zyla 5.5 sCMOS camera (Andor). Measurement of the surface area of the cell colonies, the cell-cell contact e-cadherin staining and counting of the nuclei were performed with ImageJ 1.51j8 software (National Institute of Health). Graphic representations and statistical analyses were generated using Prism 5 (Graphpad Software).

### Immunoblotting

The following antibodies were used for immunoblotting: anti-phospho AKT pS473 (Cell Signalling Technology, Cat. No. 4051S), anti-pan-AKT (Cell Signalling Technology, Cat. No. 9272S), anti-AKT1 and AKT2 (sc-5270 and sc-1618 respectively, Santa Cruz Biotechnology) and anti-GAPDH (Thermo Fischer Scientific, Cat. No. MA5–15738).

### Cell migration and invasion

Four thousand cells were seeded in 20 μL hanging drops of DMEM supplemented with 7.5% FCS to obtain spheroids. After 7 days, they were transferred in DMEM-3% FCS supplemented with 1 μg/mL Matrigel (Corning Inc) and cultured for 8 days. Pictures were taken with an AMG Evos microscope 40× objective (Thermo Fisher Scientific Inc) and spheroid surface areas were measured using ImageJ 1.51j8 software (National Institute of Health).

### Electrical cell impedance measurements

Electrode-containing arrays 8W10E+ (Ibidi, Cat. No.72040) were equilibrated with 400 μL of DMEM-7.5% FCS for 1 h on the ECIS Z Theta apparatus (Ibidi). 200 μL of medium was then removed and replaced by 200 μL of cell suspension containing 250.000 cells +/− 10 μM MK-2206 (Selleck Chemicals, Cat. No.S1078), 10 μM Rapamycin (Selleck Chemicals, Cat. No.S1039) or 10 μM Erlotinib (Selleck Chemicals, Cat. No.S7786). Electrical properties of the cells were then measured for 15 h. Raw data were exported from the ECIS software and used to generate graphical representations with the Prism software (Additional files 1, 2, 3, 4, 5, 6, 7, 8 and 9). Phenotypic differences were determined via analyses over a 4 h period of the most robust increase in electrode resistance (measured at 4000 Hz) during the cell attachment phase (Additional file 9).

### Cell viability and proliferation assays

The viability of CAL33 cells was measured after 48 h of cell culture +/− 10 μM MK-2206 or 5 μM Rapamycin with an Adam apparatus (NanoEntek) according to the manufacturer’s instructions. For proliferation assays, 25,000 cells were seeded in duplicate in 60 mm diameter dishes in DMEM-7.5% FCS. Twenty-four hours later, medium was replaced with DMEM-7.5% FCS in the presence or absence of MK-2206 (5 μM) or Rapamycin (5 μM). Cells were counted with a Z Series Coulter (Beckman Coulter Life Sciences) on day 3 and 4.

### Statistical methods

Statistical analyses presented in the figures were performed on at least 3 independent replicates. One-way ANOVA with Bonferroni’s post-test was performed using GraphPad Prism v5.03 (GraphPad Software) to determine significance of the observed effects.

## Results

### Inhibition of AKT1 induces morphological changes in CAL33 cells

**Fig. 1:**
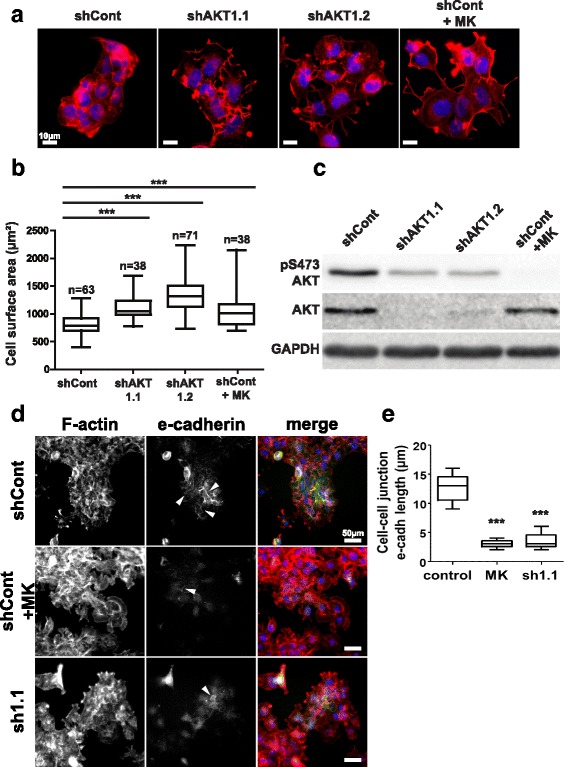
AKT1 inhibition induces CAL33 cell spreading. **a** Alexa555-phalloidin (red) staining of the actin cytoskeleton in CAL33 cells expressing a control shRNA (shCont), two independent shRNA sequences targeting AKT1 (shAKT1.1 and shAKT1.2) or control cells treated with the pan-AKT inhibitor MK-2206 (shCont+MK). Nuclear DNA was counterstained with Hoechst 33,342 (blue). **b** Average cell surface areas were measured by dividing the surface of cell colonies by the number of nuclei in the colonies. **c** AKT activity and expression levels were evaluated by immunoblot with an anti-phospho-AKT antibody (pS473-AKT) and an anti-pan-AKT antibody. GAPDH was used as a loading control. **d** Immunostaining of e-cadherin (green) and Alexa555-phalloidin (red) staining of the actin cytoskeleton (F-actin) in CAL33 cells expressing a control shRNA (shCont), an shRNA sequences targeting AKT1 (sh1.1) or control cells treated with the pan-AKT inhibitor MK-2206 (shCont+MK). Nuclear DNA was counterstained with Hoechst 33,342 (blue). White arrows indicate examples of cell-cell junction e-cadherin staining. **e** Measurements of the mean length of cell-cell junctional e-cadherin per cell. Box-and-whisker plots presented in the figure extend from the 25th to 75th percentiles with whiskers displaying the whole range of the dataset and horizontal bars representing the median. The number of measurements from at least three independent experiments is displayed above each plot; one-way ANOVA with Bonferroni’s post-test: *** *p* < 0.001 as compared to shCont

Inhibition of AKT activity in the PI3K/AKT constitutively active CAL33 cell line was performed with either MK-2206, a pharmacological inhibitor that targets AKT isoforms 1–3 or with two independent AKT1-specific shRNA sequences. Both methods of AKT inhibition resulted in strong alterations of cellular morphology as revealed by staining of the actin cytoskeleton with Alexa555-coupled phalloidin (Fig. 1a). CAL33 cells increased spreading, formed looser cell-cell contacts and exhibited a significantly increased surface area when AKT was inhibited (Fig. 1b). Decreased gene expression levels of AKT1 by two independent shRNA sequences specific to AKT1 strongly reduced both total AKT protein expression and AKT activity as revealed by reduced phosphorylation at the S473 site (Fig. 1c). Similarly, cells treated with the AKT inhibitor experienced an even stronger reduction of AKT phosphorylation (Fig. 1c). Moreover, western blots performed with antibodies specific to either AKT1 or AKT2 revealed that AKT1 is the major isoform expressed in the CAL33 cells (Additional file 10: Figure S1). The observed morphological changes induced by the inhibition of AKT in CAL33 cells are typical of a loss of epithelial phenotype. We observed disruption of the epithelial marker e-cadherin expression and membrane localization in these conditions, suggesting the occurrence of an EMT-like process (Fig. 1d and e and Additional file 11: Figure S2). Combined, these results indicate that AKT1 activity is necessary to maintain CAL33 cells epithelial morphology.

### Inhibition of AKT1 increases invasion capacity of CAL33 cells

**Fig. 2:**
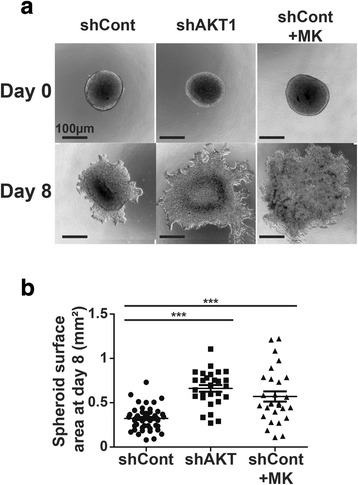
AKT1 inhibition increases CAL33 cell migration in a 3D assay. **a** Spheroids generated with CAL33 cells expressing a control shRNA (shCont), a shRNA sequence targeting AKT1 (shAKT1) or control cells treated with the pan-AKT inhibitor MK-2206 (shCont+MK) were embedded in an extracellular matrix. Pictures of the spheroids were acquired at day 0 and day 8 for analysis. **b** Spheroid surface area was measured after 8 days. Error bars represent the data mean +/− SEM from at least three independent experiments; one-way ANOVA with Bonferroni’s post-test: *** *p* < 0.001 as compared to shCont

The increased spreading of the CAL33 cells induced by AKT1 inhibition suggested that their invasive capacities may be increased as well under these conditions. We tested this hypothesis in a 3D spheroid invasion assay in purified extracellular matrix (Matrigel, Corning). Inhibition of AKT1 via both shRNA and pharmacology strongly increased cell spreading from the spheroids into the extracellular matrix after eight days (Fig. 2a and b) thus suggesting an increased cellular ability to invade and migrate through a matrix.

### Electrical property-based cell phenotype screening

**Fig. 3:**
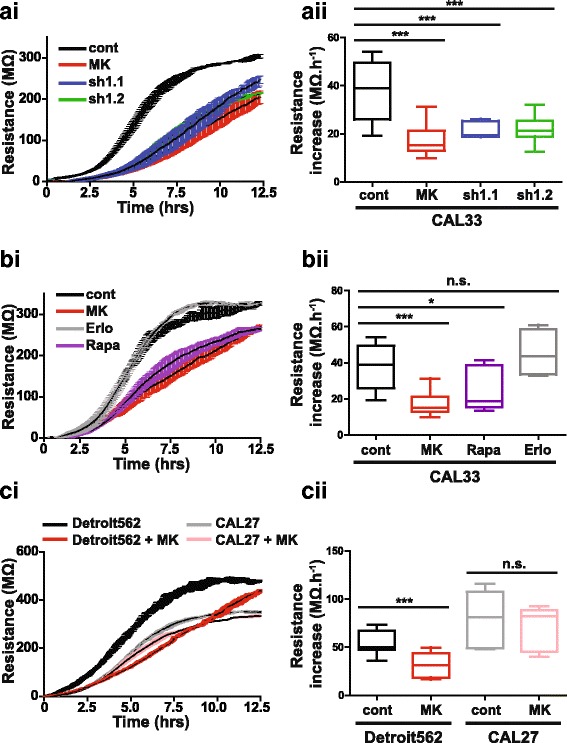
AKT1 inhibition modifies cellular electrical properties. **ai**, **aii** Electrode resistance measured at 4000 Hz for CAL33 cells expressing a control shRNA (cont), two independent shRNA sequences targeting AKT1 (sh1.1 and sh1.2) or control cells treated with a pan-AKT inhibitor (MK) was observed for up to 12.5 h (ai). The cell spreading/attachment phase was determined via calculation of the increased electrical resistance during the period of 4-8 h following cell plating (aii). **bi**, **bii** Electrode resistance measured at 4000 Hz for CAL33 cells treated with the pan-AKT inhibitor MK-2206 (MK), the mTORC1 inhibitor Rapamycin (Rapa) or the EGF receptor inhibitor Erlotinib (Erlo) was observed for up to 12.5 h (bi). Changes in electrical resistance between 4 and 8 h after cell spreading were then quantified (bii). **ci**, **cii** Electrode resistance measured at 4000 Hz for Detroit562 or CAL27 cells +/− MK-2206 (MK) was observed for up to 12.5 h (ci). As above, changes in electrical resistance between 4 and 8 h after cell spreading were then quantified (cii). Each dataset was generated from at least three independent experiments. One-way ANOVA with Bonferroni’s post-test: *** *p* < 0.001, *p < 0.05, n.s.: non-significant as compared to control

To further characterize the phenotype induced by AKT1 inhibition, we measured the electrical properties of cells (impedance, resistance and capacitance) that were seeded at high density on an electrode [32]. Time-course experiments of CAL33 monolayers revealed that treatment with MK-2206 at the time of spreading or knockdown of AKT1 significantly decreased the rate of resistance increase during the cell-adhesion phase (~ 4-8 h following cell plating, Fig. 3a). These changes are not likely to be due to cell death or proliferative defects as MK-2206 only slightly reduced cell viability at 48 h and proliferation rates were not affected by any of the treatments at this time point (Additional file 12: Figure S3A and B). We then performed a phenotypic-based screening for compounds that may induce the same phenotype as the AKT inhibitor. mTOR is one of the downstream effectors of AKT [33] and the EGF receptor is an upstream activator of the PI3K/AKT pathway mutated in approximately 90% of HNSCC cases [34, 35]. We therefore used the classical mTOR targeting drug rapamycin and the EGFR inhibitor erlotinib in this screening. Rapamycin phenocopied the effect of AKT inhibition while erlotinib did not alter the electrical properties of the cells (Fig. 3b). Although erlotinib is a potent cytotoxic drug for HNSCC cell lines [35, 36], the absence of an observed phenotype indicates that this electrical screening technique can delineate specific differences between compounds with respect to morphological or cytotoxic alterations. We next used the same assay to screen additional HNSCC cell lines based on their response to AKT inhibition (Fig. 3c). Treatment of CAL27 cells with MK-2206 did not induce significant changes in their electrical properties whereas Detroit562 cells displayed a phenotypic change comparable to that of the CAL33 cells upon inhibition of AKT (Fig. 3c, Additional file 11: Figure S2 and Additional file 13: Figure S4). Taken together, our results demonstrate that the electrical phenotype screening of compounds and cell lines allows the discovery of potential treatment-induced EMT-like processes in HNSCC cell lines.

### Validation of results obtained via electrical phenotypic screening

**Fig. 4:**
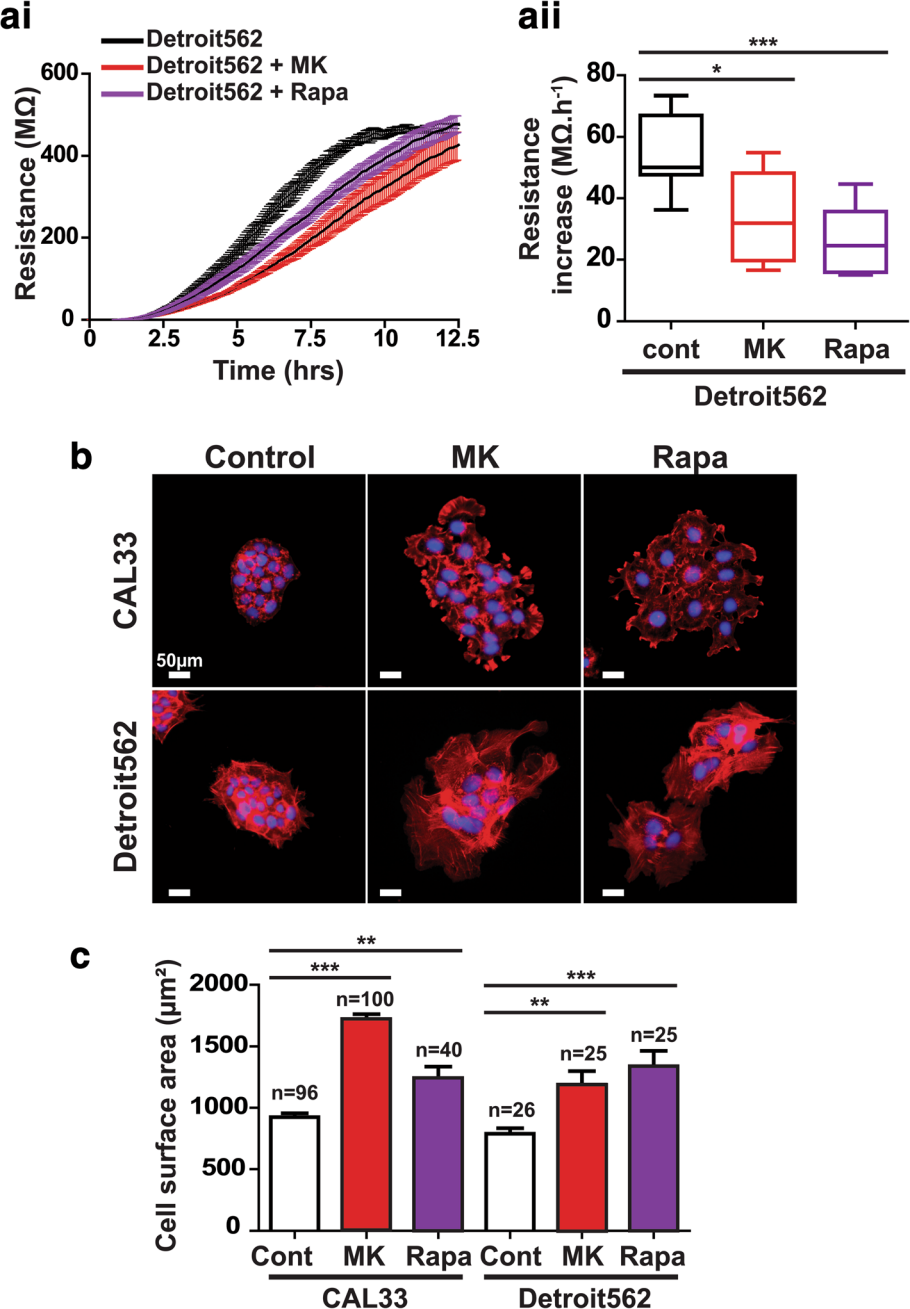
Further experiments validating the use of electrical properties screening for uncovering of phenotypic differences. **ai**, **aii** Electrode resistance measured at 4000 Hz for Detroit562 cells treated with the pan-AKT inhibitor MK-2206 (MK) or the mTORC1 inhibitor Rapamycin (Rapa) was measured for up to 12.5 h (ai). Combined values for the resistance increase from 4 to 8 h after cell spreading (aii). **b** Staining of the actin cytoskeleton with Alexa555-phalloidin (red) for CAL33 and Detroit562 cells treated with either MK-2206 (MK) or Rapamycin (Rapa). Nuclear DNA was counterstained with Hoechst 33,342 (blue). **c** Average cell surface areas were measured by dividing the surface of cell colonies by the number of nuclei in the colonies. The number of measurements from at least three independent experiments is displayed above the histograms. Data represent mean +/− SEM, one-way ANOVA with Bonferroni’s post-test: *** *p* < 0.001, ** *p* < 0.01, *p < 0.05 as compared to control

In order to confirm the results described above, we assessed the ability of rapamycin to induce a phenotypic change in Detroit562 cells. Electrical resistance increase was strongly impaired with both the AKT inhibitor MK-2206 and the mTOR inhibitor rapamycin (Fig. 4a). This result is consistent both with the ability of MK-2206 to induce a phenotypic change in Detroit562 cells and the ability of rapamycin to induce comparable changes in CAL33 cells (Fig. 2b and c). Finally, in order to confirm the correlation between the electrical property modifications and morphological alterations, cells were stained with Alexa555-coupled phalloidin to observe the actin cytoskeleton of both CAL33 and Detroit562 cells treated with either MK-2206 or rapamycin (Fig. 4b). Quantitative validation of our phenotypic screening approach showed that cell spreading of both cell lines was significantly increased upon treatment with the two compounds (Fig. 4c).

## Discussion

Although the AKT family comprises three isoforms, most studies only consider global AKT1–3 activity. However, recent investigations have now elegantly revealed the specific effects mediated by AKT1 and AKT2 isoforms in both the ErbB2-driven and the polyoma middle T (PyMT)-driven mammary adenocarcinoma transgenic mouse models [26, 27, 37]. In these models, constitutive activation of AKT1 in the mammary epithelium accelerated the onset of tumorigenesis but drastically decreased the number of metastatic lesions [27]. Conversely, removal of the *akt1* gene strongly delayed the onset of tumorigenesis [37]. Furthermore, expression of a constitutive active form of AKT2 had no effect on tumor onset but strongly increased the occurrence of lung metastases [26]. Combined, these results suggest that AKT1 and AKT2 may play opposite roles in the metastatic process and that differential AKT isoform activities require further consideration in cancer studies. The relevance of these findings in mouse models have been recently reported for human breast tumors [29, 30]. Gene expression datasets obtained from breast cancer cell lines and clinical samples revealed a strong association between high *akt1* expression, low expression of mesenchymal markers and better patient survival. Collectively, these results strongly suggest that AKT1 activity promotes early stages of tumorigenesis but restricts the tumor cell metastatic potential. However, these results have never been extended to non-breast cancer models. Our study suggests that AKT1 specific activity is also involved in the maintenance of the epithelial phenotype of HNSCC cells. An important implication is that AKT1 may also be predictive of the invasive capacities and aggressiveness of HNSCCs. Enhanced AKT/mTOR activity is common in oral carcinomas [38] and alterations of the PI3K/Akt/mTOR pathway are found in a large majority of HNSCCs [39]. As the consensus from the literature is that these pathways promote cell survival and metastasis, a great effort has been placed on pharmacological targeting of the PI3K pathway in HNSCC [34, 40]. The majority of previous in vitro studies on HNSCCs have focused on classical readouts such as association of AKT activity with cell survival and lower sensitivity to radiotherapy and chemotherapy [41–44]. Other research has indicated that increased AKT activity may promote a mesenchymal phenotype [45]. However, none of the previous in vitro (or in vivo) studies on HNSCCs have considered the influence that specific AKT isoform expression could have on the outcome of AKT inhibition. Here we have observed that in certain subtypes of HNSCCs, which predominantly express AKT1 in comparison to AKT2, AKT1 inhibition leads to a more invasive phenotype. Therefore, it appears that, as has been recently revealed in an extensive body of work for breast cancer [26, 27, 29, 30], additional cancer types such as HNSCC may require AKT isoform analysis to predict the outcome of pan-AKT inhibitors. Despite encouraging results obtained with mTOR inhibitors [46–48], most of the clinical trials involving agents targeting the PI3K/Akt/mTOR pathway have failed to pass phase II. Consistently, a phase II clinical trial with the pan-AKT inhibitor MK2206 on recurrent and metastatic HNSCC resulted in a partial response and was not moved to phase III so far (ClinicalTrials.gov identifier NCT01349933). A significant consideration is that treatments targeting the PI3K or all AKT isoforms may promote the invasive capacities of cancer cells in some cases. These counterintuitive results may explain why PI3K and AKT inhibitors are not included yet in clinical practices [49]. The possibility that some targeted therapies may increase the invasive and thus metastatic potential despite a reduction of the tumor burden should therefore be scrutinized.

We described a screening technique to determine potential pro-metastatic effects of a drug by electrical properties measurements. This method is mostly used for established models such as maintenance of endothelial cell-cell junctions or wound healing on confluent cell monolayers [50, 51]. Components of the electrical changes observed during the early phase of cell attachment can be more difficult to confidently interpret. Linking such electrical measurements to a given biological effect therefore requires complementary experiments. We observed that the increase of electrical resistance during the attachment phase is lower when AKT1 is inhibited in some but not all HNSCC cell lines. Conventional morphology and invasion assays indicated that these differences in electrical properties correlated with cell spreading, decreased cell-cell contact and acquisition of an invasive phenotype. We thus envision this technique becoming a valuable tool for high-throughput screening of drug-induced metastatic potential.

Alterations in the PI3K/AKT/mTOR pathway occur in 38% of all cancers as demonstrated on nearly 20,000 tumors of diverse origins [52]. More specifically in HNSCCs, pooled results from several databases showed that the PIK3CA gene, encoding the p110alpha catalytic subunit of the PI3K, is amplified in approximately 70% of HNSCC cell lines and 20% of HNSCCs clinical cases analyzed [53]. These results pinpoint the importance of the PI3K/AKT pathway dysregulation in cancers. The CAL33 and Detroit562 cell lines that adopted a mesenchymal phenotype upon AKT or mTORC1 inhibition bear a H1047R activating mutation in PIK3CA. Conversely, the CAL27 cell line does not bear this mutation and does not display comparable modifications in the same experimental conditions. Maintenance of the epithelial phenotype of CAL33 and Detroit562 cells could rely on the constitutive activity of the PI3K/AKT1 axis that is absent in CAL27 cells. In this case, the presence of both AKT1 amplification and constitutive PI3K activity would be a prognostic marker for pro-metastatic effects of pan-AKT inhibitors.

## Conclusions

In summary, we extended the role of AKT1 specific activity in the maintenance of an epithelial phenotype to a new cancer type. This suggests that the differential role of AKT isoforms may be widespread in cancer. Furthermore, we have established an electrical screening protocol that correlates with metastatic cellular phenotypes. We believe that our method will be valuable for future efforts involving high-throughput screening of new pharmacological compounds and for the detection of potential deleterious effects of drugs that are currently approved for clinical use. Finally, future efforts are required to delineate specific functions of AKT isoforms to avoid unwanted outcomes from the use of pan-AKT inhibitors or to develop specific AKT2/3 inhibitors.

## Additional files

Additional file 1: CAL33-shControl cells treated with Erlotinib, Rapamycin and MK-2206 electrical resistance measurements. Raw output file of the ECIS measurement of resistance in MΩ at a frequency of 4000 Hz. (XLS 432 kb)

Additional file 2: CAL33-shControl cells untreated or treated with Rapamycin and MK-2206 electrical resistance measurements. Raw output file of the ECIS measurement of resistance in MΩ at a frequency of 4000 Hz. (XLS 213 kb)

Additional file 3: CAL33-shControl cells untreated or treated with MK-2206 and CAL33-shAKT1.1 and 1.2 cells electrical resistance measurements. Raw output file of the ECIS measurement of resistance in MΩ at a frequency of 4000 Hz. (XLS 147 kb)

Additional file 4: CAL33-shControl cells untreated or treated with MK-2206 and CAL33-shAKT1.1 and 1.2 cells electrical resistance measurements. Raw output file of the ECIS measurement of resistance in MΩ at a frequency of 4000 Hz. (XLS 220 kb)

Additional file 5: CAL33-shControl cells untreated or treated with MK-2206 and CAL33-shAKT1.1 and 1.2 cells electrical resistance measurements. Raw output file of the ECIS measurement of resistance in MΩ at a frequency of 4000 Hz. (XLS 86 kb)

Additional file 6: Detroit562 and CAL27 cells untreated or treated with MK-2206 electrical resistance measurements. Raw output file of the ECIS measurement of resistance in MΩ at a frequency of 4000 Hz. (XLS 1380 kb)

Additional file 7: Detroit562 cells untreated or treated with MK-2206 or Rapamycin electrical resistance measurements. Raw output file of the ECIS measurement of resistance in MΩ at a frequency of 4000 Hz. (XLS 227 kb)

Additional file 8: Detroit562 cells untreated or treated with MK-2206 or Rapamycin electrical resistance measurements. Raw output file of the ECIS measurement of resistance in MΩ at a frequency of 4000 Hz. (XLS 213 kb)

Additional file 9: Electrical data used to generate the figures. The ECIS measurements of resistance in MΩ at a frequency of 4000 Hz were normalized to the first measurement and plotted in the Graphpad Prism software to generate the traces shown in Figs. 3a-c and 4a. The quantification data were obtained by measuring the mean resistance increase during the cell attachment phase (from 4 to 8 h after cell spreading). (XLSX 140 kb)

Additional file 10: **Figure S1.** AKT1 and AKT2 isoform expression in CAL33, Detroit562 and CAL27 cells. AKT1 and AKT2 expression levels were evaluated by immunoblot with specific anti-AKT antibody in CAL33 cells expressing a control shRNA (shCont), two independent shRNA sequences targeting AKT1 (sh1.1 and sh1.2) and in Detroit562 and CAL27 cells. GAPDH was used as a loading control. (PDF 26 kb)

Additional file 11: **Figure S2** Analysis of e-cadherin expression and localization by immunofluorescence in CAL33 cells. Immunostaining of e-cadherin (green) and Alexa555-phalloidin (red) staining of the actin cytoskeleton (F-actin) in CAL33 cells expressing a control shRNA (shCont), an shRNA sequences targeting AKT1 (sh1.2) or control cells treated with the pan-AKT inhibitor MK-2206 (MK), Rapamycin (Rapa) or Erlotinib (Erlo). Nuclear DNA was counterstained with Hoechst 33,342 (blue). (PDF 1545 kb)

Additional file 12: **Figure S3** Cell viability and proliferation assays. (A) The viability of CAL33 cells expressing a control shRNA (CAL33), two independent shRNA sequences targeting AKT1 (shAKT1.1 and shAKT1.2) or treated with the pan-AKT inhibitor MK-2206 (MK) or the mTORC1 inhibitor Rapamycin (Rapa) was measured after 48 h. Statistical analysis was performed using one-way ANOVA with Bonferroni’s post-test: *** *p* < 0.001, n.s.: non-significant. (B) CAL33 cell proliferation assays of the same experimental manipulations as described in part (A). Cell proliferation is represented as a fold-increase over the starting number of cells and was measured after 3 and 4 days of treatment. (PDF 44 kb)

Additional file 13: **Figure S4** Analysis of e-cadherin expression and localization by immunofluorescence in CAL27 and Detroit562 cells. Immunostaining of e-cadherin (green) and Alexa555-phalloidin (red) staining of the actin cytoskeleton (F-actin) in CAL27 and Detroit562 cells treated with the pan-AKT inhibitor MK-2206 (MK), Rapamycin (Rapa) or Erlotinib (Erlo). Nuclear DNA was counterstained with Hoechst 33,342 (blue). (PDF 247 kb)

## Abbreviations

DMEM: Dulbecco’s modified Eagle’s medium
ECIS: Electrical impedance cell sensing
EGFR: Epithelial growth factor receptor
EMT: Epithelium-to-mesenchyme transition
FCS: Fetal calf serum
GAPDH: Glyceraldehyde 3-phosphate dehydrogenase
HNSCC: Head and neck squamous cell carcinoma
mTOR: mammalian target of rapamycin
PFA: paraformaldehyde
PI3K: Phosphoinositide 3-kinase
PyMT: Polyoma middle T
shRNA: short hairpin ribonucleic acid
