# Supplementary figures and images for: AKT1 restricts the invasive capacity of head and neck carcinoma cells harboring a constitutively active PI3 kinase activity
Source: BMC Cancer. 2018 Mar 5;18:249. doi: 10.1186/s12885-018-4169-0 (PMC5836445; doi:10.1186/s12885-018-4169-0)

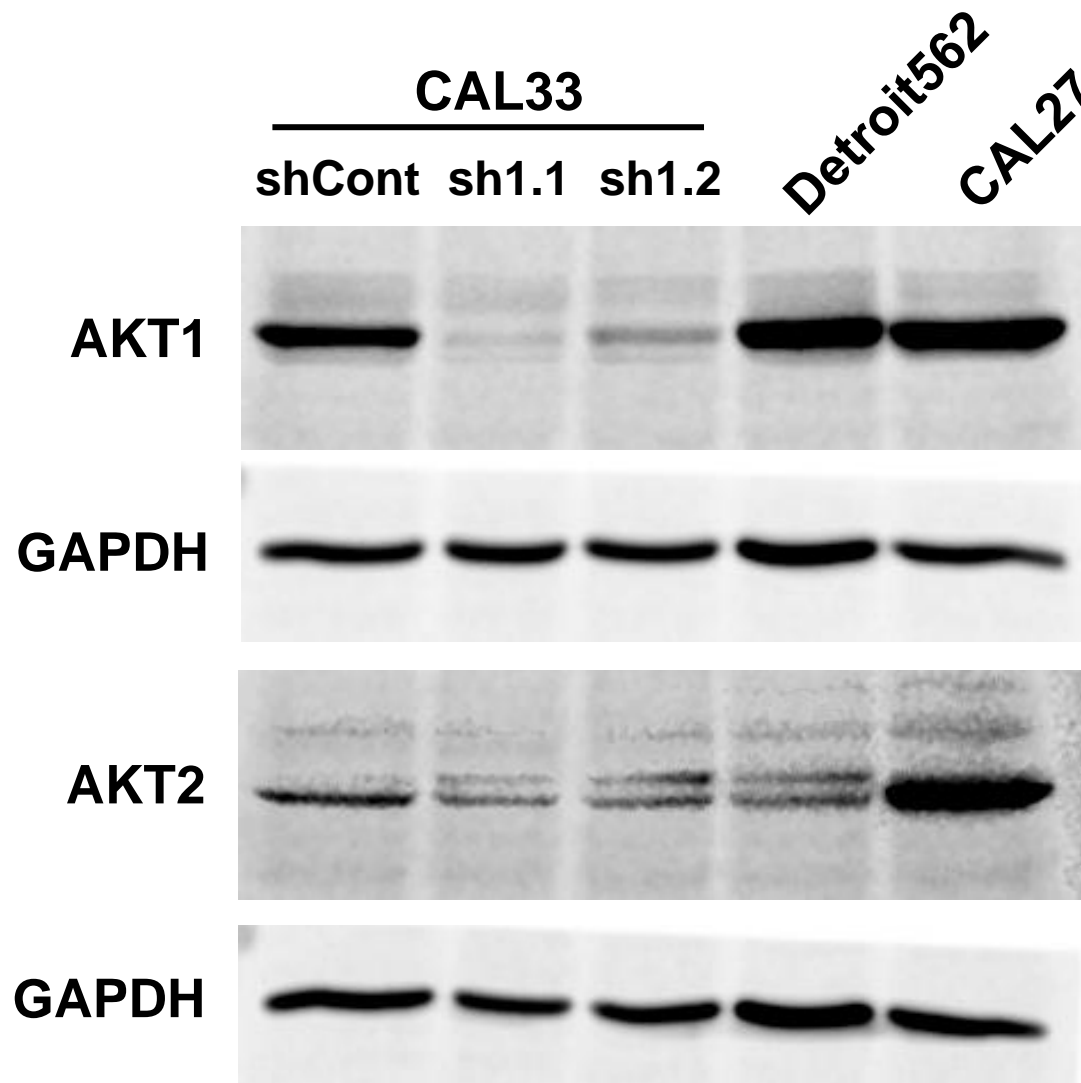

**Figure S1. Broluh, Parks et al.**

Supplement: Supplementary file 10 — Figure S1. AKT1 and AKT2 isoform expression in CAL33, Detroit562 and CAL27 cells. AKT1 and AKT2 expression levels were evaluated by immunoblot with specific anti-AKT antibody in CAL33 cells expressing a control shRNA (shCont), two independent shRNA sequences targeting AKT1 (sh1.1 and sh1.2) and in Detroit562 and CAL27 cells. GAPDH was used as a loading control. (PDF 26 kb) [file 12885_2018_4169_MOESM10_ESM.pdf]

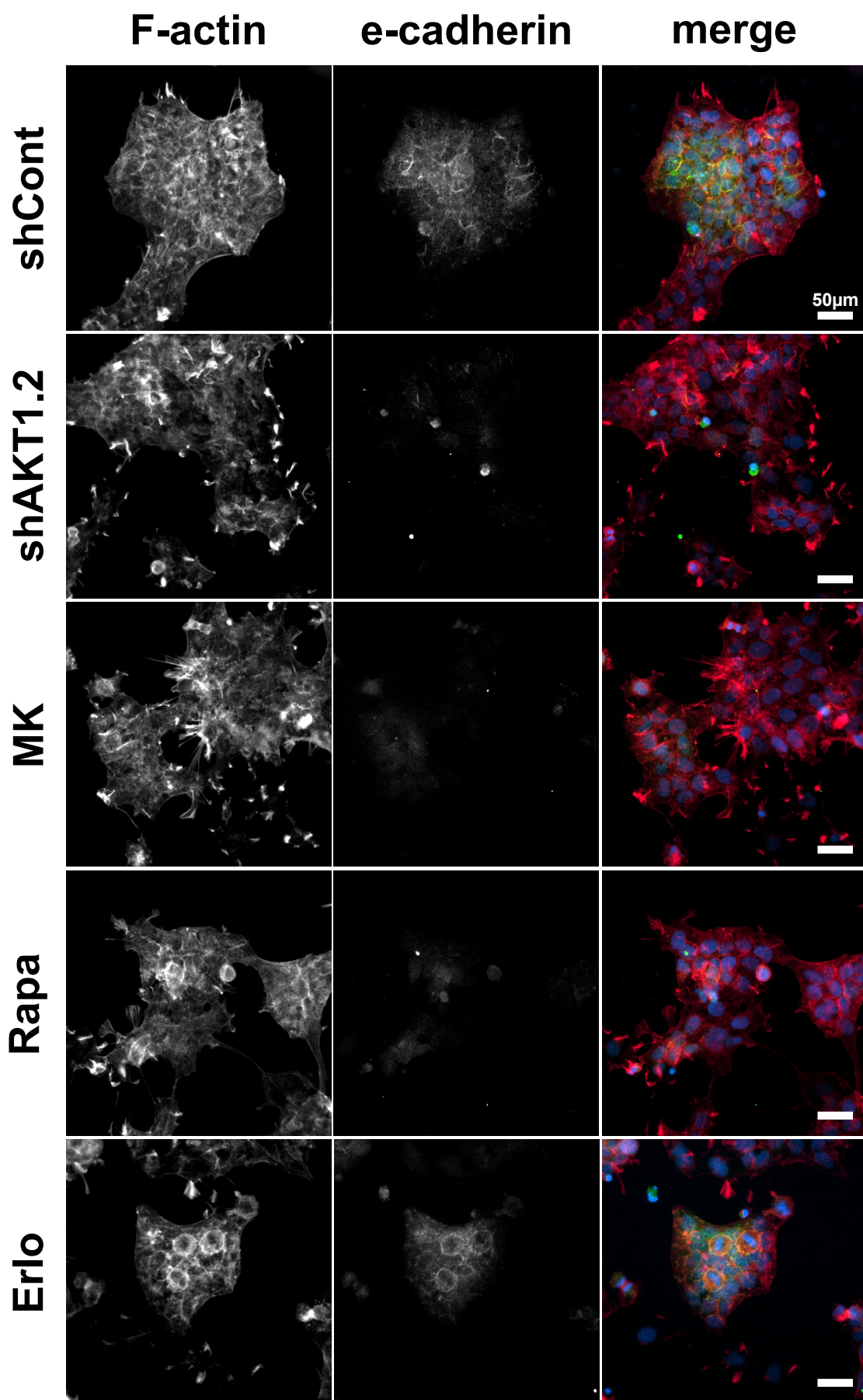

**Figure S2. Brolih, Parks et al.**

Supplement: Supplementary file 11 — Figure S2 Analysis of e-cadherin expression and localization by immunofluorescence in CAL33 cells. Immunostaining of e-cadherin (green) and Alexa555-phalloidin (red) staining of the actin cytoskeleton (F-actin) in CAL33 cells expressing a control shRNA (shCont), an shRNA sequences targeting AKT1 (sh1.2) or control cells treated with the pan-AKT inhibitor MK-2206 (MK), Rapamycin (Rapa) or Erlotinib (Erlo). Nuclear DNA was counterstained with Hoechst 33,342 (blue). (PDF 1545 kb) [file 12885_2018_4169_MOESM11_ESM.pdf]

A

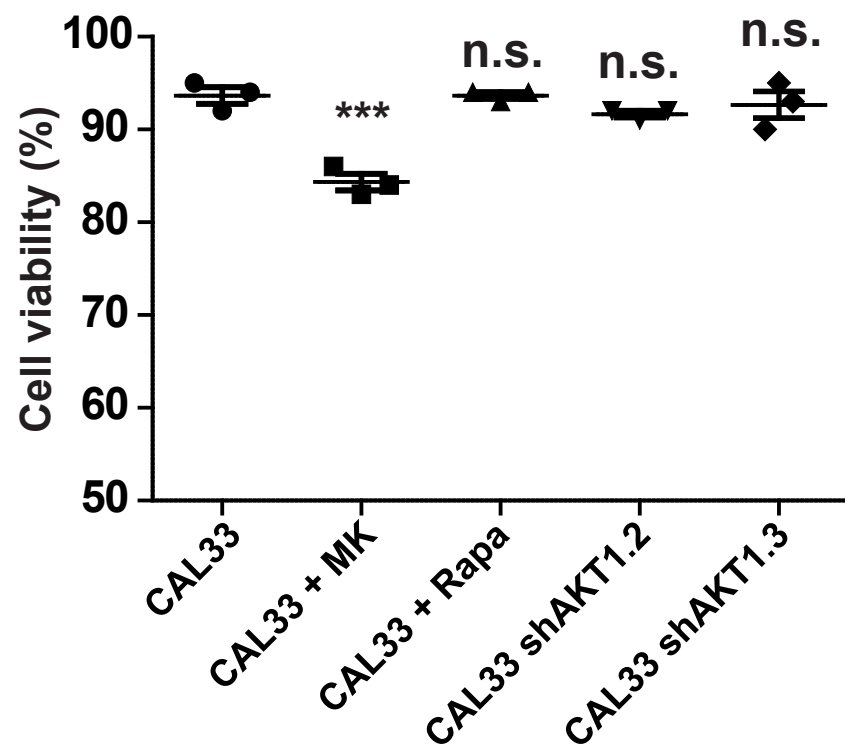

B

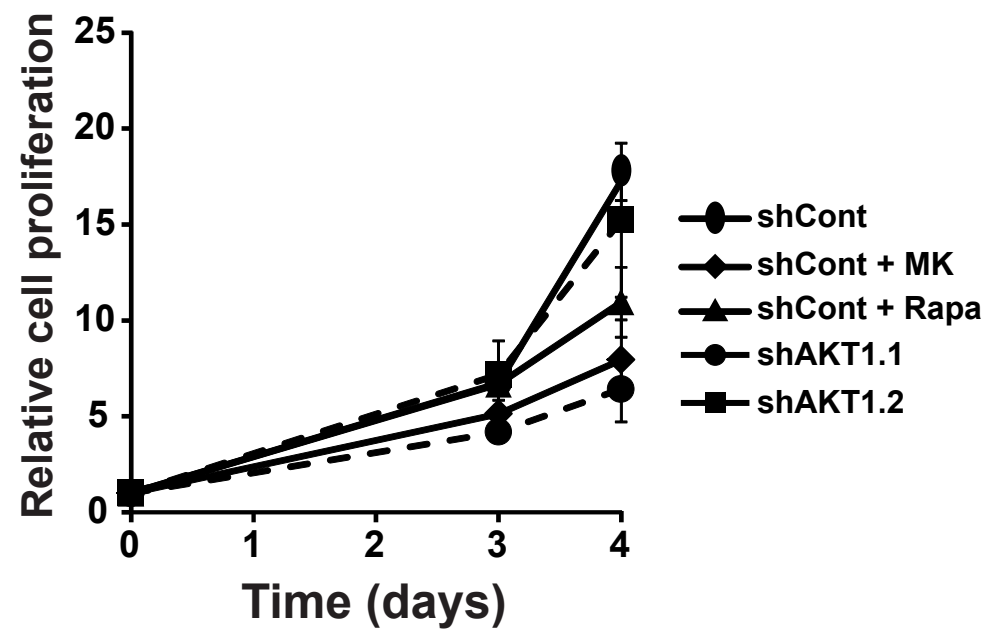

Figure S3. Brolih, Parks et al.

Supplement: Supplementary file 12 — Figure S3 Cell viability and proliferation assays. (A) The viability of CAL33 cells expressing a control shRNA (CAL33), two independent shRNA sequences targeting AKT1 (shAKT1.1 and shAKT1.2) or treated with the pan-AKT inhibitor MK-2206 (MK) or the mTORC1 inhibitor Rapamycin (Rapa) was measured after 48 h. Statistical analysis was performed using one-way ANOVA with Bonferroni’s post-test: *** p < 0.001, n.s.: non-significant. (B) CAL33 cell proliferation assays of the same experimental manipulations as described in part (A). Cell proliferation is represented as a fold-increase over the starting number of cells and was measured after 3 and 4 days of treatment. (PDF 44 kb) [file 12885_2018_4169_MOESM12_ESM.pdf]

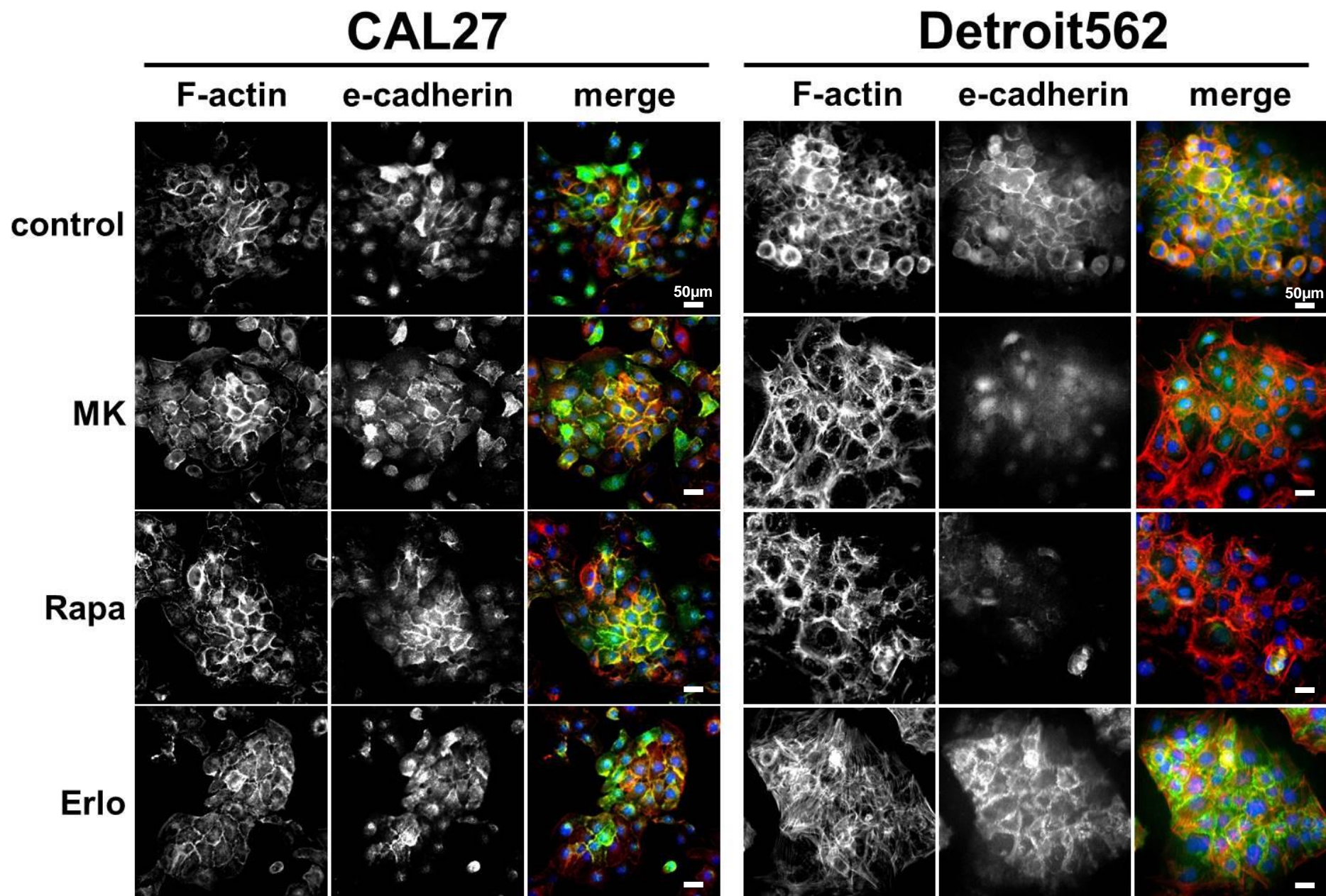

Figure S4. Brolih, Parks et al.

Supplement: Supplementary file 13 — Figure S4 Analysis of e-cadherin expression and localization by immunofluorescence in CAL27 and Detroit562 cells. Immunostaining of e-cadherin (green) and Alexa555-phalloidin (red) staining of the actin cytoskeleton (F-actin) in CAL27 and Detroit562 cells treated with the pan-AKT inhibitor MK-2206 (MK), Rapamycin (Rapa) or Erlotinib (Erlo). Nuclear DNA was counterstained with Hoechst 33,342 (blue). (PDF 247 kb) [file 12885_2018_4169_MOESM13_ESM.pdf]
